# Supplementary material for: From Quiescence to Activation: The Reciprocal Regulation of Ras and Rho Signaling in Hepatic Stellate Cells
Source: Cells. 2025 May 5;14(9):674. doi: 10.3390/cells14090674 (PMC12071349; doi:10.3390/cells14090674)
Supplement: Supplementary file 1 [file cells-14-00674-s001.zip › cells-3529301-supplementary.pdf]

Supplementary Information

From Quiescence to Activation: The Reciprocal Regulation of RAS and RHO Signaling in Hepatic Stellate Cells

Saeideh Nakhaei-Rad, Silke Pudewell, Amin Mirzaiebadizi, Kazem Nouri, Doreen Reichert, Claus Kordes, Dieter Häussinger, Mohammad Reza Ahmadian

Institute of Biochemistry and Molecular Biology II, Medical Faculty, Heinrich Heine University Düsseldorf, Düsseldorf, Germany.

Table S1. The list of primers used in this study.

Name	Sequence	Primer lenght (bp)	NCBI (RefSeq)
ERAS	Forward: CCTTGCCAACAAAGTCTAGCATC	24	NM_001434382.1
	Reverse: GCCAGCATCTTTGCATTGTGC	24	
MRAS	Forward: TGTTCCCAGTGACAACCTTCCC	24	NM_012981.3
	Reverse: GGGTCGTAGTCAGGCACGAA	24	
NOTCH1	Forward: CCCTTGCTCTGCCTAACGC	22	NM_001105721.1
	Reverse: GGGTCCTGGCATCGCTGG	22	
NOTCH2	Forward: GAGAAGAACCGCTGTCAGAATGG	24	NM_024358.2
	Reverse: GGTCGAGTATTGGCAGTCCTC	24	
HES1	Forward: CCAGCCAGTGTCAACACGA	20	NM_024360.4
	Reverse: AATGCCGGGAGCTATCTTTCT	22	
HPRT1	Forward: AAG TGT TGG ATA CAG GCC AGA	23	NM_012583.2
	Reverse: GGC TTT GTA CTT GGC TTT TCC	24	
RND3	Forward: GGAGTTTTGTCCCAATACCAAGA	24	NM_001007641.2
	Reverse: GTTCCACTAGTGTGCTGACATC	24	
RHOC	Forward: GTGGGGAATAAGAAGGACCTGAG	24	NM_001106461.1
	Reverse: GAACCGGCTCCTGTTTCATTT	24	

Table S2. Antibodies used in this study.

Antibodies	Source	Identifier	Dilution
anti-GFAP	Dako	# Z0334	WB:1/1000<br>ICC:1/500
anti-α-SMA	Dako	#M0851	1/1000
anti-ERAS	Synthetized	clone 3.5.8	1/1000
anti-p190GAP	BD Biosciences	# 610150	1/1000
anti-RHOC (c-16)	Santa cruz	# sc-12116	1/500
anti-RND3	Proteintech	# 66228-1-Ig	1/2000
anti-MRAS	Proteintech	# 14213-1-AP	1/1000
anti-NOTCH1 (D6F11) XP™	Cell Signaling	# 4380 S	1/500
anti-γ-tubulin	Sigma-Aldrich	# T5326	1/1000
anti-phospho-ERK1/2 T202/T204	Cell Signaling	# 9106	1/1000
anti-phospho-AKT S473	Cell Signaling	# 4060	1/1000
anti-phospho-AKT T308	Cell Signaling	# 2965	1/1000
anti-phospho-YAP Ser127	Cell Signaling	# 4911	1/1000
anti-YAP	Cell Signaling	# 4912	1/1000
anti-rabbit IgG Alexa Fluor 488 Conjugate	Cell Signaling	# 4412	1/500
anti-mouse IgG Alexa Fluor 555 Conjugate	Cell Signaling	# 4409	1/500
anti-mouse IgG Alexa Fluor 488 conjugated	Cell Signaling	# 4408	1/500
IRDye® 800CW Donkey anti-Rabbit IgG	LI-COR Biosciences	# 926-32213	1/5000
IRDye® 800CW Donkey anti-Mouse IgG	LI-COR Biosciences	# 926-32212	1/5000
Alexa488-conjugated goat anti-rabbit IgG	Thermo Fisher Scientific	# A11034	1/500
Alexa546-conjugated goat anti-mouse IgG	Thermo Fisher Scientific	# A4671	1/500
Alexa488-conjugated goat anti-mouse IgG	Thermo Fisher Scientific	# A11029	1/500

Table S3. Guide RNAs used in this study.

Primer 1: Target Sequenz (4):	GGCGCTCCGATAATCTCTATGGG (Exon 1 (-))
gRNA_ARG1_1-for:	TAATACGACTCACTATAGGGCGCTCCGATAATCT
gRNA_ARG1_1-rev:	TTCTAGCTCTAAAACATAGAGATTATCGGAGCGC
Primer 2: Target Sequenz (5):	TATCGGAGCGCCTTTCTCTAAGG (Exon 1 (+))
gRNA_ARG1_2-for:	TAATACGACTCACTATAGTATCGGAGCGCCTTTC
gRNA_ARG1_2-rev:	TTCTAGCTCTAAAACTAGAGAAAGGCGCTCCGAT
Primer 3: Target Sequenz (6):	GAATCTGCACGGGCAACCGGTGG (Exon 4 (+))
gRNA_ARG1_3-for:	TAATACGACTCACTATAGGAATCTGCACGGGCAA
gRNA_ARG1_3-rev:	TTCTAGCTCTAAAACCCGGTTGCCCGTGCAGATT
Primer 4: Target Sequenz (10):	TTAGTCGGTTCACAGACCTGTGG (Exon 3 (-))
gRNA_ARG1_3-for:	TAATACGACTCACTATAGTTAGTCGGTTCACAGA
gRNA_ARG1_3-rev:	TTCTAGCTCTAAAACCAGGTCTGTGAACCGACTA

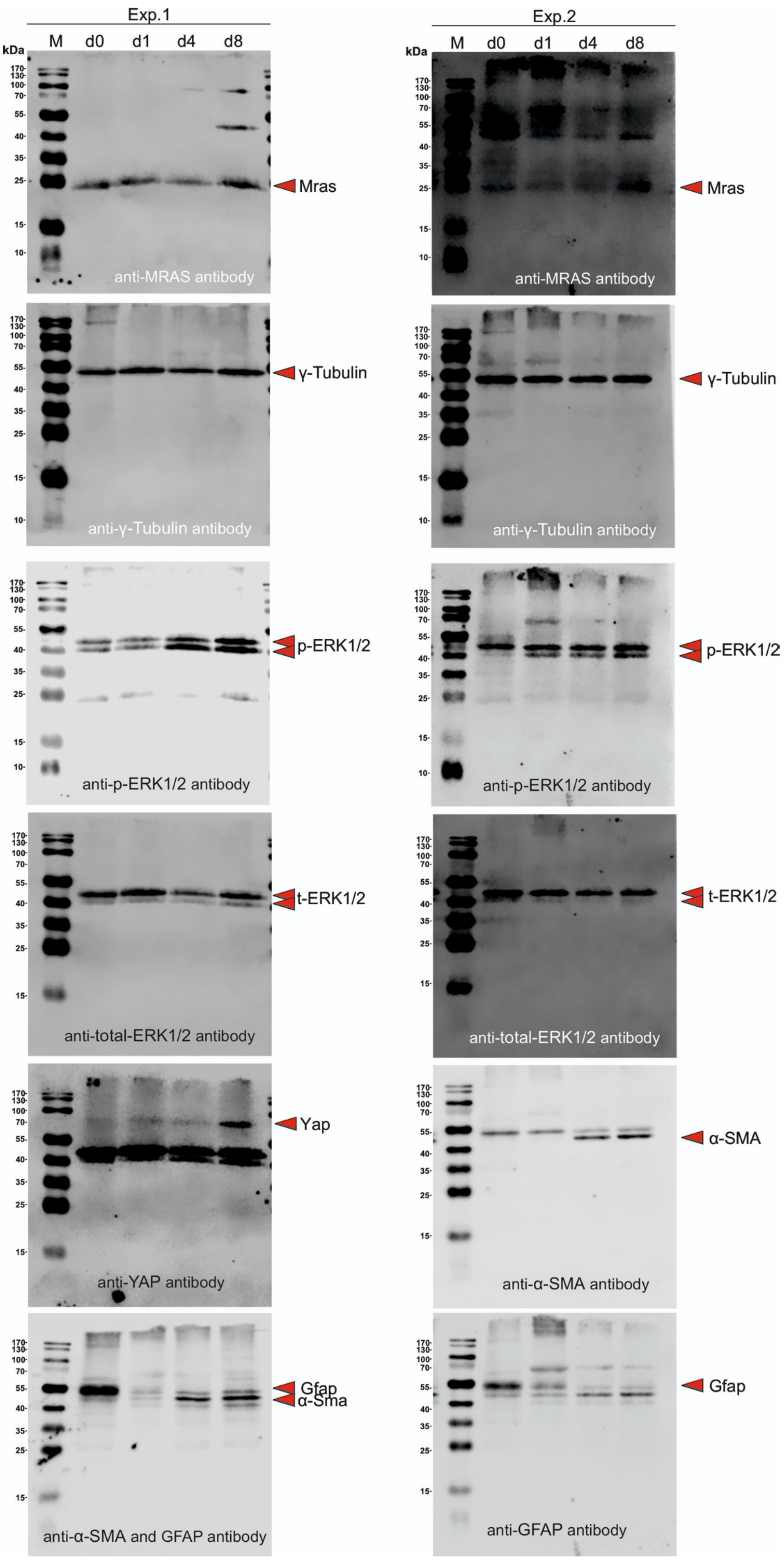
Exp.1
M d0 d1 d4 d8
kDa
Mras
anti-MRAS antibody
γ-Tubulin
anti-γ-Tubulin antibody
p-ERK1/2
anti-p-ERK1/2 antibody
t-ERK1/2
anti-total-ERK1/2 antibody
Yap
anti-YAP antibody
Gfap
α-Sma
anti-α-SMA and GFAP antibody
Exp.2
M d0 d1 d4 d8
kDa
Mras
anti-MRAS antibody
γ-Tubulin
anti-γ-Tubulin antibody
p-ERK1/2
anti-p-ERK1/2 antibody
t-ERK1/2
anti-total-ERK1/2 antibody
α-SMA
anti-α-SMA antibody
Gfap
anti-GFAP antibody

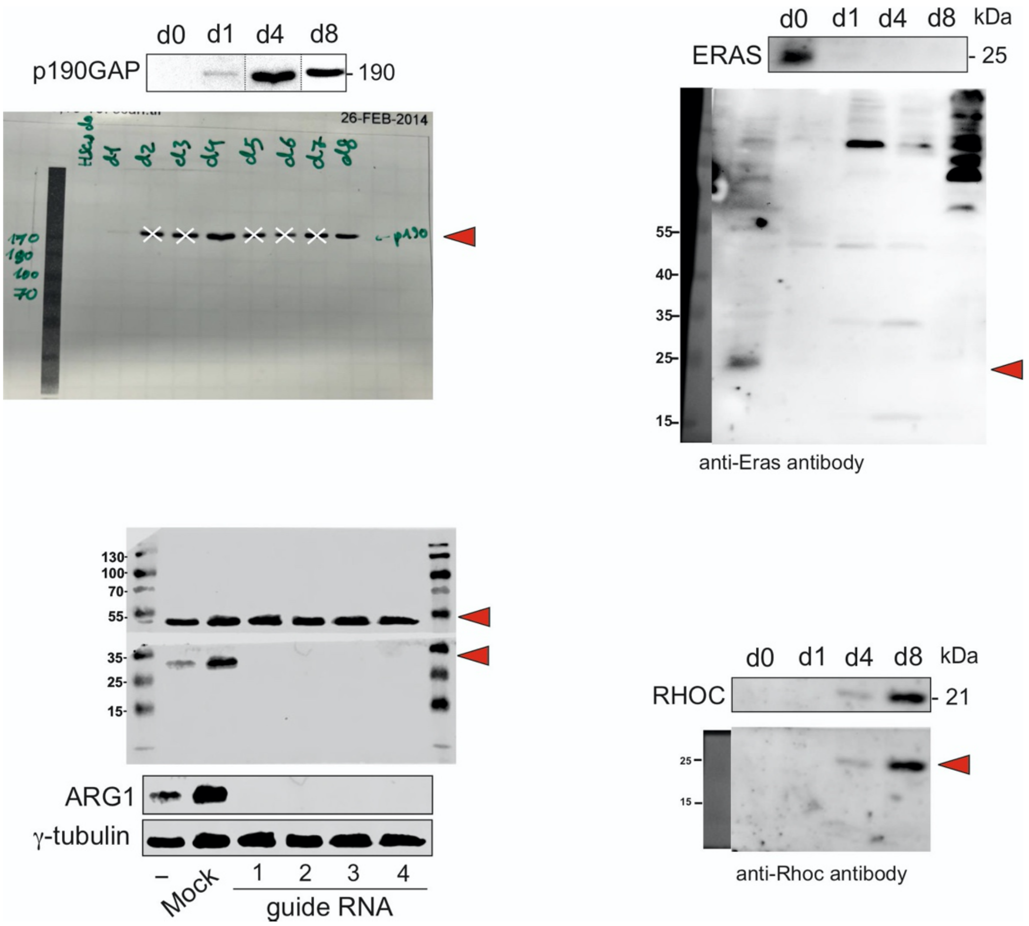

Figure S1. Original Western Blot Data. The original Western blots (n=2) are displayed below the corresponding blots presented in the main manuscript.
